# Concerted cell and in vivo screen for pancreatic ductal adenocarcinoma (PDA) chemotherapeutics

**DOI:** 10.1038/s41598-020-77373-8

**Published:** 2020-11-26

**Authors:** Somayeh Layeghi-Ghalehsoukhteh, Shreoshi Pal Choudhuri, Ozhan Ocal, Yalda Zolghadri, Victor Pashkov, Hanspeter Niederstrasser, Bruce A. Posner, Havish S. Kantheti, Ana C. Azevedo-Pouly, Huocong Huang, Luc Girard, Raymond J. MacDonald, Rolf A. Brekken, Thomas M. Wilkie

**Affiliations:** 1 Department of Pharmacology, UT Southwestern Medical Center, 6001 Forest Park Drive, Dallas, TX 75390 USA; 2 Department of Basic Science, School of Veterinary Medicine, Shiraz University, Shiraz, Iran; 3 Department of Molecular Biology and Genetics, Bilkent University, 06800 Ankara, Turkey; 4 Department of Biochemistry, UT Southwestern Medical Center, 5323 Harry Hines Blvd, Dallas, TX 75390 USA; 5 Cancer Discovery (CanDisc) Group, UT Southwestern Medical Center, 6001 Forest Park Drive, Dallas, TX 75390 USA; 6 Department of Surgery, University of Arkansas for Medical Sciences, Little Rock, AR USA; 7 Hamon Center for Therapeutic Oncology Research, University of Texas Southwestern Medical Center, Dallas, TX USA; 8 Simmons Comprehensive Cancer Center, University of Texas Southwestern Medical Center, Dallas, TX 75390 USA; 9 Department of Molecular Biology, UT Southwestern Medical Center, 5323 Harry Hines Blvd, Dallas, TX 75390 USA; 10 Department of Surgery, University of Texas Southwestern Medical Center, Dallas, TX USA

**Keywords:** Cancer therapy, Chemotherapy, Cancer models, Cancer, Pancreatic cancer

## Abstract

PDA is a major cause of US cancer-related deaths. Oncogenic Kras presents in 90% of human PDAs. Kras mutations occur early in pre-neoplastic lesions but are insufficient to cause PDA. Other contributing factors early in disease progression include chronic pancreatitis, alterations in epigenetic regulators, and tumor suppressor gene mutation. GPCRs activate heterotrimeric G-proteins that stimulate intracellular calcium and oncogenic Kras signaling, thereby promoting pancreatitis and progression to PDA. By contrast, Rgs proteins inhibit Gi/q-coupled GPCRs to negatively regulate PDA progression. Rgs16::GFP is expressed in response to caerulein-induced acinar cell dedifferentiation, early neoplasia, and throughout PDA progression. In genetically engineered mouse models of PDA, Rgs16::GFP is useful for pre-clinical rapid in vivo validation of novel chemotherapeutics targeting early lesions in patients following successful resection or at high risk for progressing to PDA. Cultured primary PDA cells express Rgs16::GFP in response to cytotoxic drugs. A histone deacetylase inhibitor, TSA, stimulated Rgs16::GFP expression in PDA primary cells, potentiated gemcitabine and JQ1 cytotoxicity in cell culture, and Gem + TSA + JQ1 inhibited tumor initiation and progression in vivo. Here we establish the use of Rgs16::GFP expression for testing drug combinations in cell culture and validation of best candidates in our rapid in vivo screen.

## Introduction

Pancreatic ductal adenocarcinoma (PDA) is the third leading cause of cancer-related deaths in the US. PDA is increasing in frequency owing to its association with obesity, smoking, and type 2 diabetes^1–5^. PDA treatment is severely compromised because most diagnoses are made late in disease progression. Surgical resection remains the most effective therapeutic strategy but is restricted to early stage diagnosis and is rarely, if ever, curative. In spite of the decades of research and recent advances, the 5-year survival rate has only improved to 9%, and less than 20% of patients survive 1 year^6^.

Current treatment options for PDA are further limited by the nature of the driver mutations. Kras oncogenic mutations (e.g. Kras^G12D^) are found in over 90% of human PDA. Chemotherapy targeting the most frequent Kras mutation found in PDA has not been achieved. Targeting the Ras pathway has been more successful but severe side effects limit effectiveness. Conventional chemotherapy and radiotherapy have marginal benefits, with minimal increase in patient survival. Gemcitabine (Gem) was introduced as a first-line therapy for advanced PDA^7^. Gem alone or in combination with other drugs, or other multidrug regimens, such as FOLFIRINOX, only modestly prolong survival^7–10^. Thus, there is a pressing need for systematic and robust screens to develop novel, effective PDA therapeutics for early stage disease and after successful surgical resection to delay or prevent relapse. Our strategy to identify better therapeutics is to screen drugs in primary cell culture, followed by validation in a mouse model of early disease progression.

Genetically engineered mouse models (GEMMs) expressing oncogenic Kras mutations have been developed to investigate PDA initiation and progression. In GEMMs, PDA initiation and maintenance is Kras-dependent^11,12^. Kras^G12D^ activity requires GDP/GTP exchange, at which point Kras^G12D^-GTP acquires constitutive activity^13,14^. Kras guanine nucleotide exchange factors (GEFs) can be activated by protein kinase receptors and G-Protein Coupled Receptors (GPCR)^15,16^.

GPCR signaling is modulated by Regulator of G protein signaling (Rgs) proteins, which accelerate the GTPase activity of Gq- and Gi-class alpha subunits^17–19^. Rgs gene expression can be induced by GPCR ligands they feedback inhibit^20,21^. We showed that a Rgs16::GFP transgene is an in vivo reporter of Kras activity in precancerous pancreatic neoplasia, including pancreatic intraepithelial neoplasia (PanIN), intraductal papillary mucinous neoplasm (IPMN), and throughout PDA tumor progression in *KIC;Rgs16::GFP* mice (p48^Cre/+^*; Kras*^*G12D/*+^*; Cdkn2a*^*f/f*^*; Rgs16::GFP*)^22^. Rgs16::GFP marks the earliest lesions, is proportional to and coincident with early tumor burden, and is expressed throughout PDA progression; importantly, Rgs16::GFP is not expressed in pancreas of healthy adult mice^22,23^. We previously demonstrated the utility of KIC;Rgs16::GFP mice for rapid in vivo drug screens^22^. A subset of human PDA tumors in the TCGA database (approximately 25%) is very similar to primary PDA cells from KIC mice characterized by whole transcriptome analysis of bulk RNAseq data^22^. Here, we used Rgs16::GFP expression in primary PDA cells in culture to identify synergistic activity of a cytotoxic drug combinations, and validate them in KIC;Rgs16::GFP mice.

Epigenetic mechanisms, in conjunction with genetic alterations, are crucial in cancer initiation and progression. There has been an increasing interest in targeting epigenetic regulators, particularly HDACs and members of BET family of bromodomain proteins, as an anticancer therapeutic strategy. Alterations in histone deacetylase (HDAC) activity occur in numerous cancers and have prompted the search for pharmacological agents capable of inhibiting these enzymes^24,25^.

HDAC inhibitors appear to be selective against tumor cells and show limited normal tissue toxicity in vivo^26–29^. Presently, there are two United States Food and Drug Administration (FDA)-approved HDAC inhibitors for anticancer therapy and more are undergoing clinical trials, including Trichostatin A (TSA). In this study, we found that TSA stimulates Rgs16::GFP expression in a dose-dependent manner in primary PDA cells in culture.

Recent research has shown that treatment with BET inhibitors, such as JQ1, decreased the growth of PDA cells^30^. When combined with TSA, we found a synergistic increase in efficacy of JQ1 induced cell death of cultured primary PDA cells. Therefore, we evaluated novel combinations of Gem, a standard-of-care PDA chemotherapeutic, with TSA and JQ1 in primary cell culture and rapid in vivo PDA therapeutic assays. Combination therapy with TSA synergistically increased the efficacy of Gem and/or JQ1 in primary cell culture. In vivo*,* the combination of GEM + TSA + JQ1 significantly reduced initiation and growth of spontaneous tumors. Here we demonstrate an effective screen for novel PDA therapeutics. First, primary PDA cells in culture are screened for small molecules that induce Rgs16::GFP expression in response to stress. Molecules that synergize with Gem, a standard-of-care cytotoxic drug are identified in cell culture viability assays. Efficacy in mice is validated in a rapid in vivo assay of PDA initiation and growth. These steps provide a quick and efficient approach for identifying new and effective therapeutic combinations to treat PDA.

## Results

Alterations in HDAC activity occur in numerous cancers and have prompted the search for pharmacological agents capable of inhibiting these enzymes^24,25^. Several studies have reported elevated expression of HDACs and BETs in PDA. HDAC1, 2, 3, 4, and 7 were reported to be upregulated in PDA, whereas HDAC 2 and 3, along with SIRT1, have been reported to be involved in cancer invasion and chemo-resistance^31–35^. Thus, we assessed the differential expression profile of all HDACs and BETs in human PDA tissue samples in the TCGA database and compared these to mouse models of caerulein-treated pancreatitis, PDA (KIC), and primary PDA cells from KIC mice.

### HDACs and BET proteins are highly expressed in human and mouse PDA

**Figure 1:**
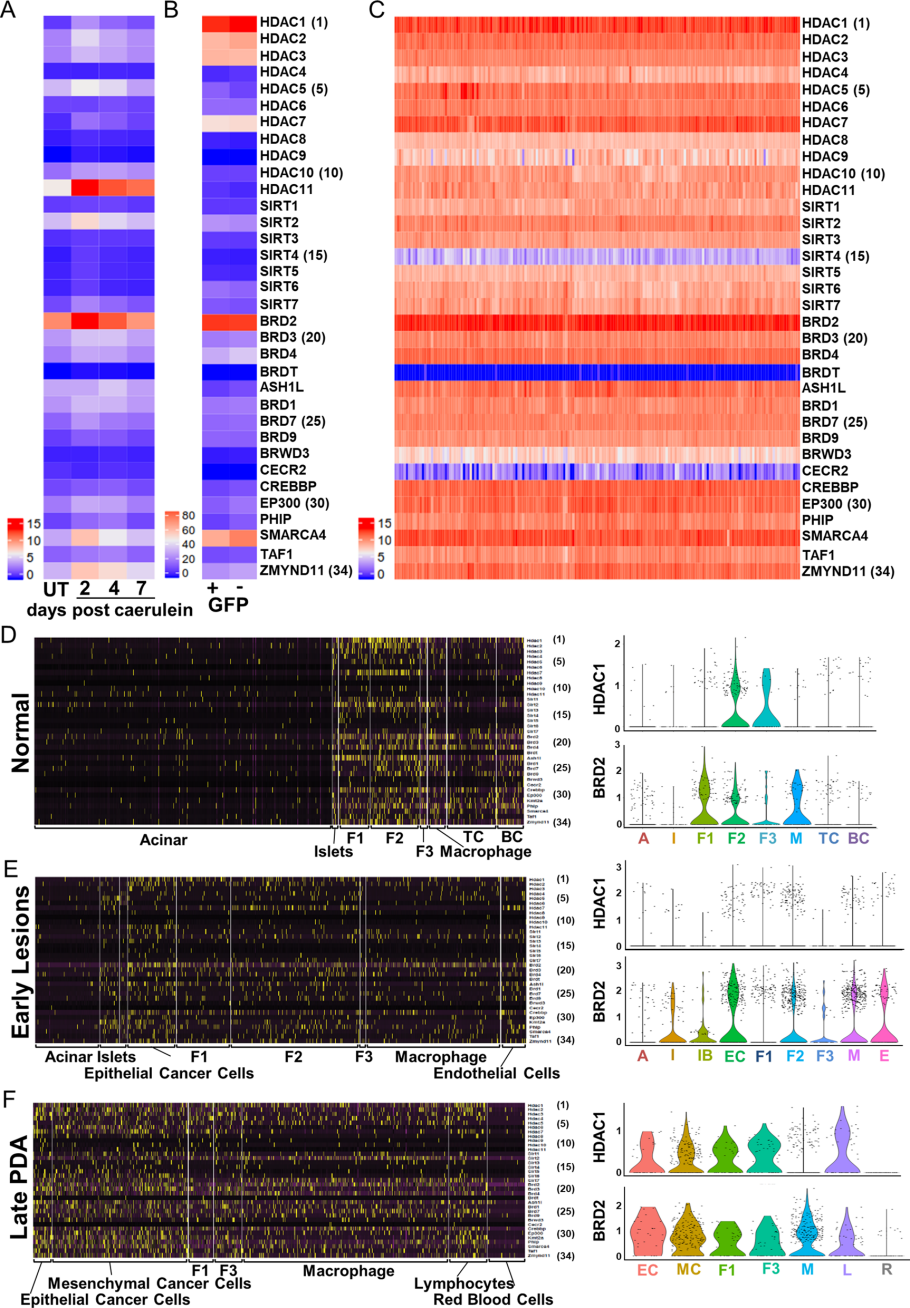
HDAC and BET family of bromodomain protein expression in caerulein induced acute pancreatitis, human PDA, and mouse PDA. Relative expression of HDACs and BET family bromodomain proteins were analyzed in (**A**) wild-type untreated (UT) pancreas and 2, 4, 7 days post caerulein injections, (**B**) mouse primacy PDA cells and, (**C**) human PDA (72 samples in TCGA database). Primary PDA cells from *KIC*;Rgs16::GFP mice (mPDAC) were treated with 50% FBS overnight and FAC sorted into GFP expressing (+) and GFP negative (−) pools. Gene expression was determined by RNA-Seq (Ocal et al., 2015). (**D**–**F**) Single-cell (sc)RNAseq profiling heat map of all cells in pancreata from (**D**) normal mice, and in *KIC* mice (**E**) early lesions and (**F**) late tumors. HDAC and BET bromodomain protein genes are differentially expressed in the identified cell populations analyzed by scRNAseq. Each column represents an individual cell, and each row is the expression value for a single gene. Genes are listed in the same order in (**A**–**E**). Violin plots show a representative HDAC (HDAC1) and BET family bromodomain protein 2 (Brd2) from each sample. Cell types are (**D**) A, acinar; I, islet endocrine cells; F1-F3, fibroblasts; M, macrophage; TC, T cells; BC, B cells. (**E**) I, islet endocrine other than beta cells; IB; endocrine beta cells; EC; epithelial cancer; E, endothelial cells. (**F**) MC, mesenchymal cancer cells; L, Lymphocytes (Treg). Analysis and figure generation were performed using R statistical software [R Core Team (2018). R: A language and environment for statistical computing. R Foundation for Statistical Computing, Vienna, Austria. URL https://www.R-project.org/].

We analyzed the differential expression of HDACs and BETs at various stages of disease progression in mice. First, we compared expression in normal untreated (UT) pancreas of adult mice to pancreata collected from mice injected (i.p.) with caerulein 2, 4, and 7 days post-treatment (Fig. 1A). Acinar-to-ductal metaplasia (ADM) is greatest at d2 post-caerulein, and morphology gradually returns to normal as the exocrine pancreas recovers by day 7^36^. Expression of nearly all HCAC and BET genes reflects this pattern, showing highest expression at day 2, sequentially declining towards normal levels at days 4 and 7.

HDAC and BET expression in early stage ADM was compared to primary PDA cells from KIC mice (Fig. 1B) and human tumor samples (Fig. 1C) in the TCGA dataset (n = 72) collected predominately from stage 1 and 2 patients. In mice, the relative expression of HDAC and BET proteins is similar, except for the reciprocal pattern of HDAC1 and HDAC11 in ADM and primary PDA cancers (Fig. 1B). Primary cancer cells isolated from KIC-Rgs16::GFP mice were sorted for high and low expression of Rgs16::GFP (2.6-fold differential expression following 12 h incubation in 50% or 5% FBS, respectively) (Fig. 1B). By contrast, in human PDA tumors, containing cancer and stromal cell types, nearly all HDACs (except SIRT4) and BETs (except BRDT, BRWD3, and CECR2) were highly expressed (Fig. 1C).

We further determined the expression of HDACs and BETs during PDA progression in KIC mice. Pancreas from normal wild type mice, 40 day old KIC (early KIC, with early neoplastic lesion initially confirmed by ultrasound and then histology), and 60 day old KIC (late KIC, with advanced tumors) were digested and processed for a single cell RNA sequencing using 10 × Genomics platform^37^. HDAC1 and BRD2 were highly expressed in PDA compared to normal pancreas, both in humans and in epithelial and mesenchymal cancer cells in KIC mice (Fig. 1D–F). HDAC overexpression has been associated with poor prognosis in PDA patients^31^.

### Regulators of histone acetylation induce Rgs16::GFP in mouse primary PDA cells cultured from KIC;Rgs16::GFP mice

Rgs16::GFP is expressed in the earliest ductal lesions and throughout PDA progression in KIC;Rgs16::GFP^22^ that expresses a constitutively active *Kras*^*G12D*^ allele and deletion of the tumor suppressor Ink4a^38^. KIC mice with homozygous deletion of *Cdkn2a* (Ink4a/Arf) develop PDA tumors by 3 weeks. Half of the mice die by 8 weeks. *P48::Cre* drives the deletion of *Cdkn2a* and activation of Kras^G12D^ in pancreas progenitor cells, and all duct, exocrine and endocrine cells in adult mice. Rgs16::GFP expression is Kras^G12D^-dependent in the earliest ductal neoplasia (P18) in KC and KIC mice^22^.

**Figure 2:**
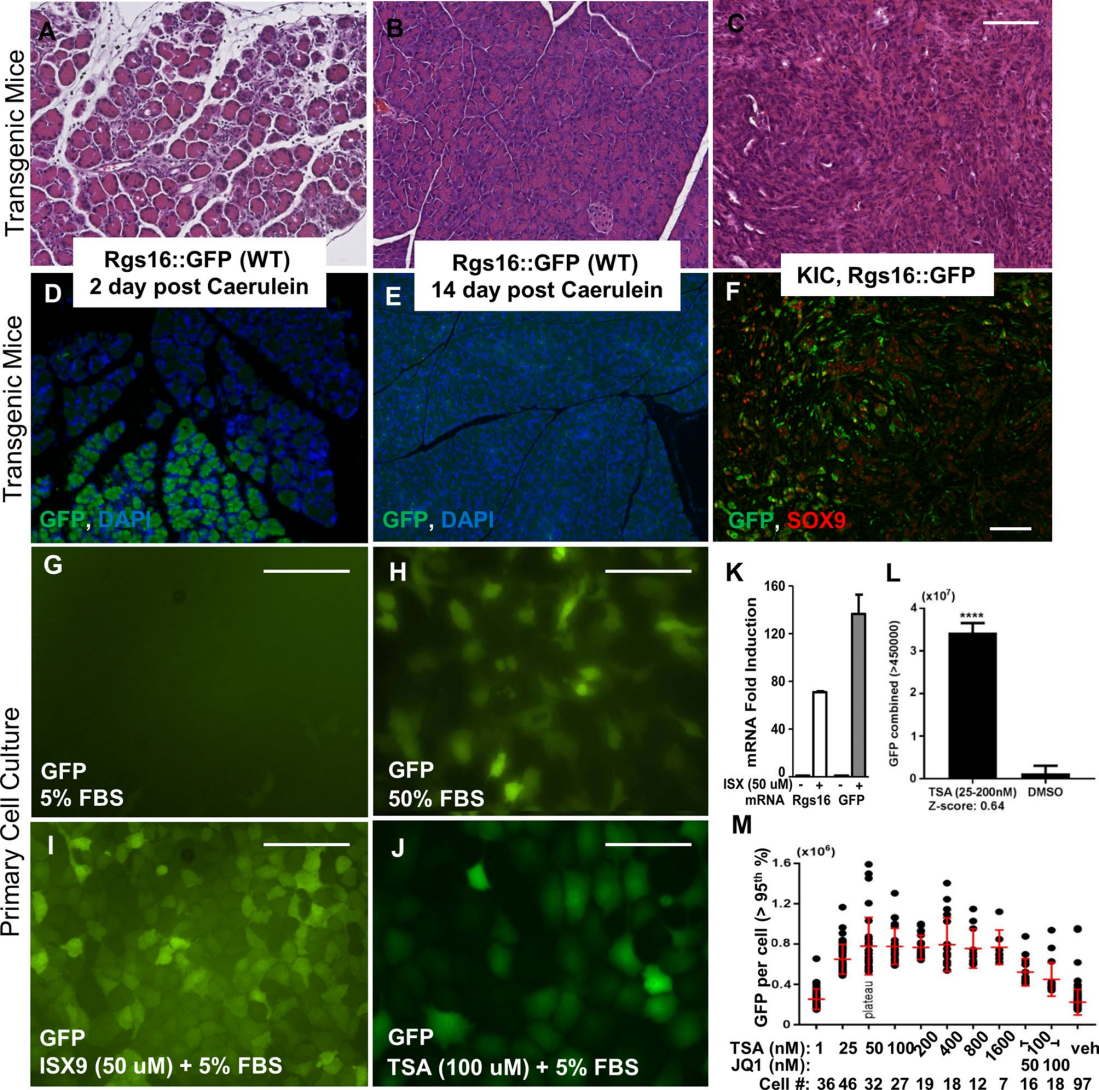
Rgs16::GFP expression is induced by HDAC and HAT inhibitors. Hematoxylin and eosin staining of pancreas sections from (**A**) Rgs16::GFP mouse 2 days post caerulein, (**B**) Rgs16::GFP mouse 14 days post caerulein, and (**C**) KIC mice tumor. Immunofluorescence showing Rgs16::GFP is expressed in pancreas of (**D**) Rgs16::GFP mouse 2 days post caerulein, but not (**E**) after 14 days post caerulein and the pancreas has healed (**F**) Rgs16::GFP is co-expressed with Sox9 in PDA cancer cells in tumors from KIC mice. (**G**) Rgs16::GFP is not expressed (basal) in PDA cells grown in 5% serum. Rgs16::GFP is induced by (**H**) 50% FBS (24 h), (**I**) ISX9 (50 uM, 5% FBS, 24 h); (**J**) TSA (100 µM, 5% FBS, 24 h). (**K**) Endogenous Rgs16 and transgenic Rgs16::GFP mRNA are induced by ISX9 (50 µM, 5% FBS, 24 h). (**L**) Total GFP fluorescence intensity in mPDA primary cells (5% FBS) in individual wells treated with TSA (25–200 nM) or vehicle (DMSO). (**M**) GFP fluorescence intensity of single mPDA cells in TSA (1–1600 nM) or TSA (100 nM) + JQ1 (50 or 100 nM). 2000 cells were plated per well, attached, incubated 12 h (5% FBS) with or without drug. GFP intensity quantitated in individual cells; top 5 percentile indicated. Peak GFP expression was 25–200 nM TSA. Cell counts decrease with increasing drug concentrations because TSA and JQ1 are cytotoxic, even after short incubation periods. Scale bars are 100 µM. Graphpad Prism v7 software[https://www.graphpad.com/ (current version); licensed] was used for data analysis and visualization.

Rgs16::GFP is not expressed in normal pancreas acinar tissue (Supplementary Fig. S1A), nor in the islets of mice with normal blood glucose^23^. Pancreatic stress provoked by caerulein injection induces a transient, gradient response of acinar cell dedifferentiation in the pancreas. Rgs16::GFP expression is induced in affected acini during the interval of one to four days after caerulein treatment (Fig. 2A,D). Rgs16::GFP expression completely disappears by day 14 as the pancreas heals (Fig. 2B,E).

In KC or KIC mice, Rgs16::GFP is expressed throughout PDA progression, marks the earliest lesions, is only expressed in areas of neoplasia, and is proportional to early tumor burden in KIC;Rgs16::GFP mice (Supplementary Fig. S1)^22^. Immunofluorescence analysis of KIC;Rgs16::GFP tumors show GFP is co-expressed with Sox9 positive cancer cells (Fig. 2C,F). Additionally, we have previously shown that orthotopic transplantation of primary cancer cells isolated from KIC;Rgs16::GFP PDA tumors regrow GFP-positive PDA specifically in duct-like structures in recipient NOD-SCID mice^22^. Primary PDA cells in culture do not express Rgs16::GFP when grown in media with 5% FBS (Fig. 2G), but Rgs16::GFP expression is induced within 16 h of treatment with 50% FBS (Fig. 2H). The HAT activator ISX9^39^ induces Rgs16::GFP protein in approximately 90% early passage mouse primary PDA cells (50 uM ISX9, 5% FBS; Fig. 2I), and mRNA of the endogenous Rgs16 gene and the Rgs16::GFP transgene within 16 h of treatment (Fig. 2K).

Histone deacetylase inhibitors (HDACi) such as TSA^40^ are cytotoxic for pancreatic cancer cell lines and inhibit growth of different cancer types in vivo^41–43^. TSA is a potent inducer of Rgs16::GFP in primary PDA cells (Fig. 2J,L), and induced GFP in a dose dependent manner (Fig. 2M). Since BET bromodomain proteins are readers of histone acetylation, we assayed JQ1, a selective inhibitor of BET with efficacy against a number of different cancers, but it did not induce Rgs16::GFP in cultured primary PDA cells (5% FBS). Gem, standard of care chemotherapeutic for PDA, also did not induce Rgs16::GFP (data not shown).

### TSA and JQ1 synergistically inhibit growth in mouse primary PDA cells

**Figure 3:**
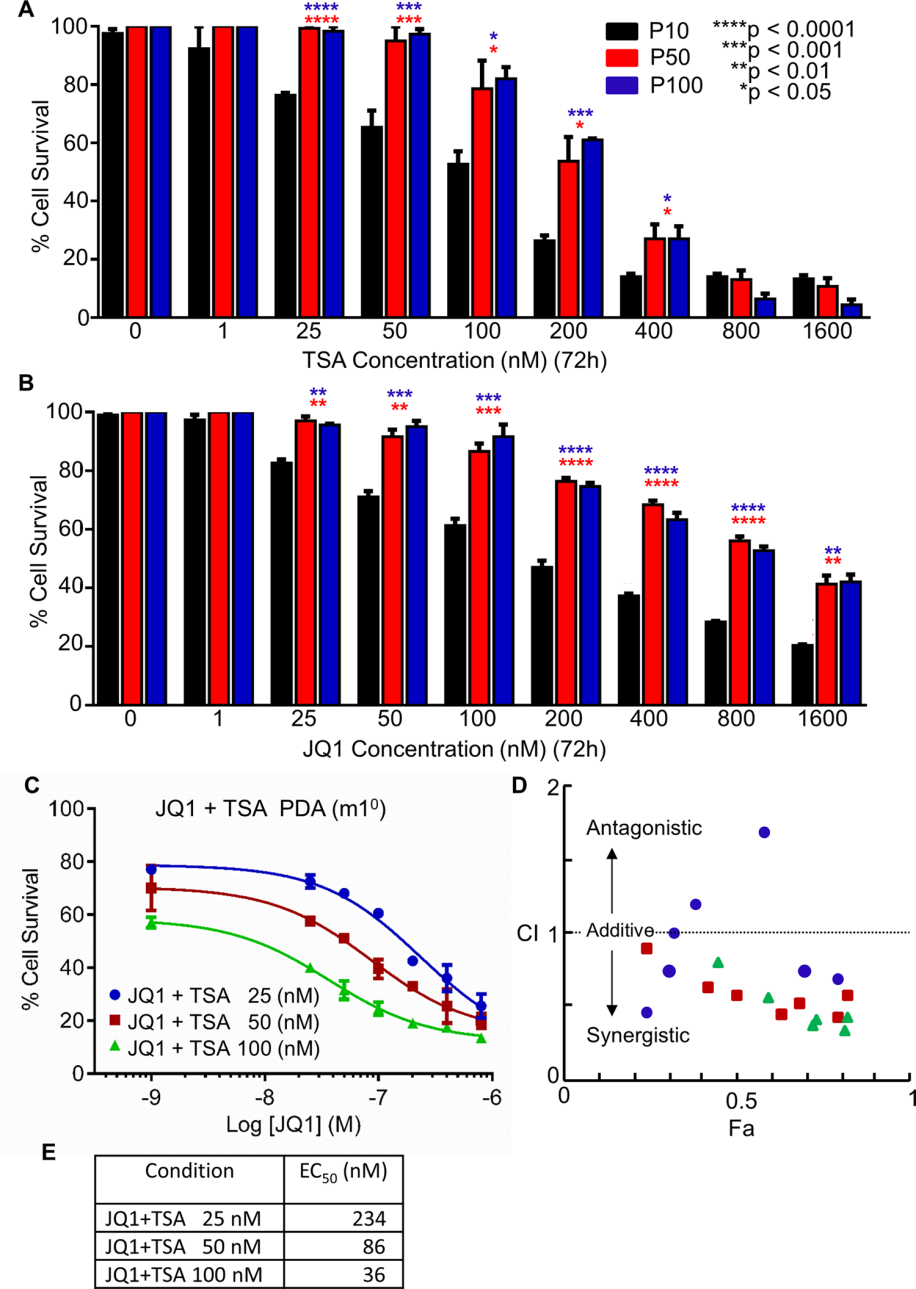
HDAC inhibitor (TSA) and BRD4 inhibitor (JQ1) are potent suppressors of early passage PDA primary cells. Primary mouse PDA cells of increasing passage number (10, 50, 100) were treated with increasing concentrations (1, 25, 50, 100, 200, 400, 800, 1600 nM) of (**A**) TSA or (**B**) JQ1 for 72 h. Cell viability was measured by Resazurin sodium salt assay. Data are expressed as Mean ± SEM from three independent experiments. (**C**) Primary mouse PDA cells were treated with increasing concentrations of JQ1 at three concentrations of TSA (25, 50, or 100 nM) for 72 h. Cell viability at sub uM concentrations of JQ1 significantly decreased with increasing [TSA]. Data are shown as Mean ± SEM from two independent experiments. (**D**) Combination index (CI) analysis showed synergistic effects for combined TSA + JQ1. The line at CI = 1 indicates additivity; data points below indicate synergy. Synergy with JQ1 increased at higher concentrations of TSA. (**E**) EC_50_ of JQ1 at three concentrations of TSA (25, 50, or 100 nM). Graphpad Prism v7 software [https://www.graphpad.com/ (current version); licensed] and CompuSyn (https://www.combosyn.com/; publicly available) were used for data analysis and visualization.

HDAC inhibitors and other regulators of epigenetic modifications are cytotoxic for tumor cells and show low toxicity in vivo. TSA stimulated dose-dependent cell death of PDA primary cells in culture (Fig. 3A). Likewise, JQ1 stimulated PDA cell death in a dose-dependent manner (Fig. 3B). Both TSA and JQ1 had greater cytotoxicity in low passage primary PDA cells (P10) than high passage cells (P50 or P100) (Fig. 3A,B; all subsequent studies were done in low passage cells). Additionally, high passage P100 cells did not induce Rgs16::GFP in response to TSA, ISX9, or high serum. Regardless of passage number, TSA was more effective than JQ1 in inhibiting cell growth.

BET inhibitors retard pancreatic ductal adenocarcinoma cell proliferation and enhance Gem cytotoxicity^35^. However, BET therapeutics are often limited by acquired drug resistance^44^. Therefore, we evaluated the synergistic activity of TSA and JQ1 in low passage primary PDA cells. TSA synergizes with JQ1 to potently suppress primary PDA cell growth in a dose-dependent manner (p < 0.0001) as shown in response curve and CI plot (Fig. 3C–E).

### TSA synergistically potentiates JQ1 and Gem lethality in vitro and in vivo

**Figure 4:**
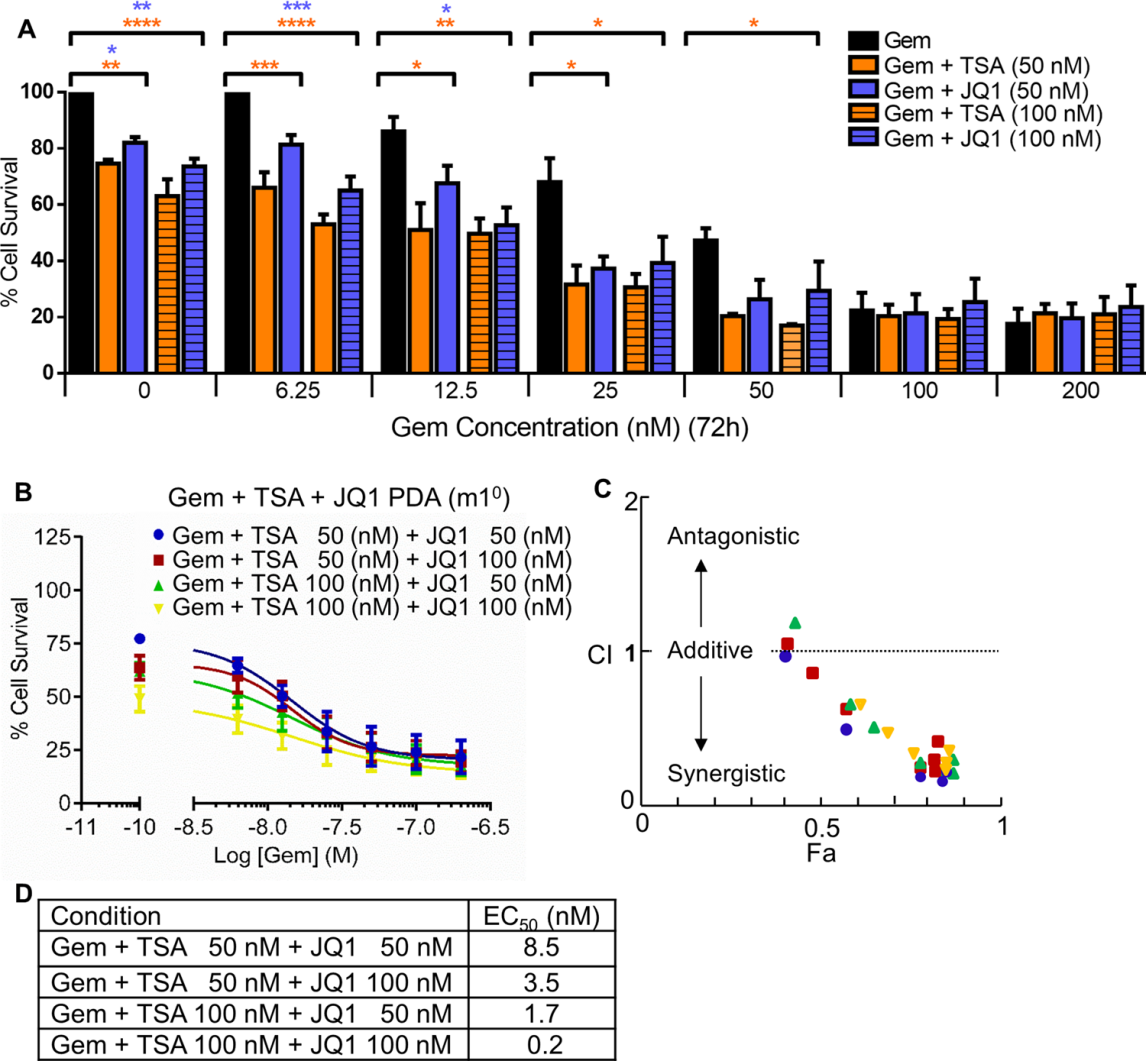
Gemcitibine (Gem) cytotoxicity in PDA primary cells is potentiated by combined TSA and JQ1. (**A**) Primary mouse PDA cells were treated with Gem alone or increasing concentrations of Gem and two concentrations (50 or 100 nM) of TSA or JQ1 or (**B**) combination of Gem + TSA + JQ1 for 72 h. Cell viability was measured by Resazurin sodium salt assay. Data are shown as Mean ± SEM from three independent experiments. (**C**) Combination index (CI) analysis was performed to identify synergistic effects for the drug combinations in (**C**). The dotted line at CI = 1 indicates additivity; data points below indicate synergy. (**D**) EC_50_ of Gem at different concentrations of TSA + JQ1. Graphpad Prism v7 software (https://www.graphpad.com/ (current version); licensed) and CompuSyn (https://www.combosyn.com/; publicly available) were used for data analysis and visualization.

**Figure 5:**
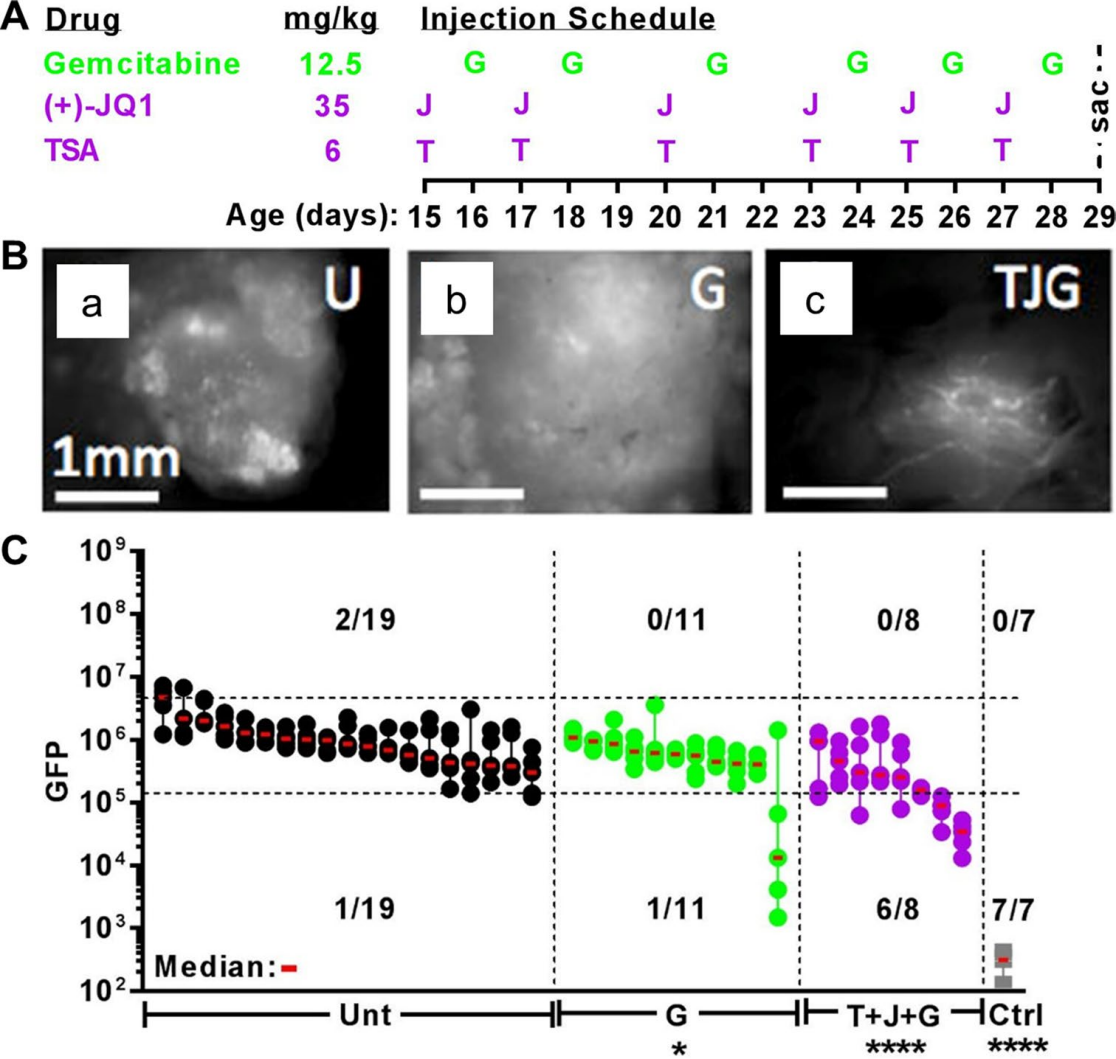
PDA growth in vivo dramatically suppressed by Gem combined with HDAC and BRD4 inhibitors (TSA + JQ1). (**A**) Injection schedule of KIC;Rgs16::GFP mice treated with Gem, Trichostatin-A (TSA) and Bromodomain inhibitor (+)-JQ1 in the Rapid in vivo Assay (RIVA) is shown with corresponding daily dosages. All mice were sacrificed at P29. (**B**) Fluorescence microscopy of tumor burden in dissected pancreata from KIC;Rgs16::GFP mice at P29 of untreated (U) or treated with Gem (G) alone or in combination (TJG) with TSA and JQ1. (**C**) RIVA quantitative comparison of tumor burden of KIC;Rgs16::GFP mice at P29 in untreated (Unt, black dots) mice or treated with Gem_1 (G, green dots) alone or in combination (T + J + G, purple dots) with TSA (T) and JQ1 (J). Control (Ctrl, grey dots) mice are non-tumorigenic Rgs16::GFP mice. Tumor burden of each mouse is represented by five non-overlapping micrographs, depicted as dots, lined vertically from the highest to the lowest GFP fluorescence intensity quantified by NIH ImageJ (https://imagej.nih.gov/ij/; publicly available). Third highest (median) intensity micrograph of each mouse is marked with a red horizontal line. Pancreata are aligned from left to right in descending medians, each treatment group separated by vertical dashed lines. Upper and lower horizontal dashed lines represent the 95th and 1st %tile of all untreated micrograph values. Number of mice with micrographs above the 95th and below the 1st %ile is shown for each group above the total sample size. Control group has only one micrograph value per pancreas. Graphpad Prism v7 software (https://www.graphpad.com/ (current version); licensed) and NIH Image J (https://imagej.nih.gov/ij/; publicly available) were used for data analysis and visualization^60–62^.

Gem is a standard-of-care chemotherapy but is only marginally effective for PDA treatment in humans^7^ or mice^22^. Because combination therapy is an effective approach, we tested the cytotoxic effect of Gem in combination with TSA and/or JQ1. Gem is potentiated by both TSA and JQ1. However, Gem in combination with TSA was more effective compared to JQ1 (Fig. 4A). In our in vitro screening, the combination of Gem + TSA + JQ1 is one of the most potent cytotoxic cocktails we have identified (Fig. 4B–D). Therefore, we tested Gem + TSA + JQ1 in our rapid, two-week in vivo assay of PDA initiation and growth (Fig. 5A–C; see Ref.^22,45^ for comparisons to Gem + Abraxane and related myosin inhibitors). Gem + TSA + JQ1 significantly decreased the initiation and progression of PDA in all KIC;Rgs16::GFP mice tested at P29, compared to untreated or Gem alone (Fig. 5). Three of 8 mice that were treated with TSA + JQ1 + Gem had the lowest tumor burden we have ever observed in KIC mice at P29.

## Discussion

Effective therapeutics and early detection strategies are needed to enhance the long-term survival of patients with PDA. A particular challenge is to identify critical signaling pathways that drive PDA formation and progression. Gem is a standard-of-care drug but is only marginally effective for PDA treatment. Combination therapy is one approach to improve treatment. We describe a concerted approach using Rgs16::GFP expression in cell culture and a rapid in vivo assay (RIVA) in a mouse model of PDA to identify improved therapeutics.

Signaling through GPCRs, which represents the largest sector of pharmaceutical development, is an attractive area that is understudied in pancreatic cancer. The long-term objective of this work is to identify therapeutics that act synergistically to inhibit the growth of mutant Kras-dependent PDA. Mutant alleles of Kras (such as Kras^G12D^) are found in more than 90% of human PDA samples^46^. Kras GEFs can be activated by protein kinase receptors and GPCR^47^. GPCR signaling can be modulated by Rgs proteins, which accelerate the GTPase activity of Gq- and Gi-class alpha subunits^17,18^. GPCR signaling can induce Rgs gene expression as a feedback regulatory mechanism^18^.

In KIC;Rgs16::GFP mice, GFP is induced in the earliest pancreatic lesions and in a subset of PDA cells throughout tumor progression^22^. In these mice, at two weeks of age, every cell in the pancreas expresses Kras^G12D^ but only a few foci have undergone acinar to ductal metaplasia (ADM) and express Rgs16::GFP^22^. Simply expressing Kras^G12D^ is not sufficient to induce acinar to ductal metaplasia (ADM) and Rgs16::GFP. However, Kras^G12D^ increases the probability that acinar cells will dedifferentiate and release inflammatory cytokines that promote further activation of oncogenic Kras^48,49^. Kras and the master regulatory transcription factor Ptf1a oppose each other’s activity. Ptf1a maintains terminally differentiated and functional acinar cells whereas oncogenic Kras promotes ADM and suppresses Ptf1a expression. At later times in tumor progression, Rgs16::GFP expression is restricted to neoplasia in PDA (Fig. 2; Supplementary Fig. S1). Thus, Rgs16 expression can be effectively used to follow tumor progression in the mouse models of PDA therapy and identify potential new therapeutics.

Primary PDA culture cells, obtained from KIC;Rgs16::GFP mice, express Rgs16::GFP and retain the capacity to form GFP + tumors when injected into recipient mice^22^. They display the morphological and gene expression profile of well differentiated epithelial cancer cells^22^. Ductal expression in adult animals appears to recapitulate early embryonic expression of Rgs16 in progenitor cells of the pre-pancreatic bud and epithelium of the developing pancreas and the earliest response to diabetic hyperglycemia^22,23^.

In primary PDA cells, Rgs16::GFP expression was induced by serum or two regulators of histone acetylation (ISX9, TSA) (Fig. 2G–M). Concentration-dependent FBS induction of Rgs16::GFP is not likely to be cell proliferation dependent as primary PDA cells have a consistent doubling time of about 12 h at FBS concentrations of 10% and above. FBS likely contains GPCR ligands that induce Rgs16::GFP gene expression in a concentration-dependent manner.

HDAC and BET family proteins are highly expressed in human PDA and mouse models of PDA, and inhibitors of these epigenetic regulators have some therapeutic value^26,30^. Treatment with the Brd4 inhibitor JQ1 alone is also not very effective^50,51^ but has shown improvement in combination with Gem. We tested inhibitors of HDAC and BET proteins alone and in combination for their cytotoxic effects on primary PDA cells isolated from KIC;Rgs16::GFP mice. TSA was the most potent inducer of Rgs16::GFP in primary mouse PDA cells in culture, but was not a particularly effective cytotoxic agent by itself. The time course of TSA induction of Rgs16::GFP expression and subsequent cell death suggested that stressed primary PDA cells expressed Rgs16::GFP 12–24 h prior to death.

Therefore, we tested whether TSA would potentiate cytotoxic drugs used to treat PDA. Indeed, TSA acted synergistically with Gem and JQ1 to kill primary PDA cells during three days of treatment in culture (Fig. 4). Low passage primary PDA cells were more responsive than high passage cells for Rgs16::GFP expression and sensitivity to cytotoxic drugs (Fig. 3) with the exception that the highest concentrations of TSA killed all cells in cultures of high passage PDA cells that had undergone crisis (P50 and P100, Fig. 3A). Because higher passage cells are more resistant to drugs, future studies could investigate if TSA sensitizes cells that had developed resistance to Gem.

Rgs16 expression in mouse PDA tumors and primary cancer cells in culture is inducible, not constitutive, in cells that express oncogenic Kras. To extend this analysis, we surveyed Rgs16 expression compared to Kras oncogenic allele status in bulk RNAseq obtained from established cancer cell lines from human PDA and a variety of other human cancer cell lines (Supplementary Table S1), and human PDA tumors (TCGA) (Supplementary Figs. S2, S3). There was no correlation between Rgs16 expression and Kras allele status in 45 human PDA cell lines, but there were only 4 WT cell lines available for comparison. Therefore, we extended the analysis to other cancers with more WT Kras alleles, and again there was no correlation. These results are all from stable cell lines, whereas Rgs16 was induced in low passage primary PDA cells, but not after these cells were passaged 100 times (Fig. 3). The capacity for Rgs16 induction was probably lost in human cancer cell lines that were passaged innumerable times. By contrast, the seven TCGA tumor samples with highest Rgs16 expression all harbored oncogenic Kras alleles (Supplementary Fig. S2, S3) and were among the clades most related to mouse PDA cells by whole transcriptome analysis^19^. Thus, primary cell culture is optimal for using Rgs16 gene expression to screen for novel therapeutics.

In summary, we have developed an innovative methodology of Rgs16::GFP reporter gene expression to identify new PDA chemotherapy in mouse primary PDA cells and validate their efficacy in vivo^22^. The validation step in Rgs16::GFP mice is quantitative, cost effective, and rapid—10 min per mouse to quantitate tumor burden following a 2 week in vivo assay prior to weaning in a spontaneous model of PDA in transgenic mice. Furthermore, the initial screen in cultured primary cells can be used to identify and characterize new GPCRs and ligands or adapted for high throughput screens to identify new chemo-preventative therapy for early neoplastic lesions in high risk patients and/or adjuvant chemotherapy after successful resection. The combined approach of HTS and in vivo validation using our Rgs16::GFP reporter is a powerful innovation in pancreatic cancer research. In future studies, we will characterize the biological mechanism of GPCR antagonists and other drugs that inhibit PDA initiation and progression in KIC mice. This work suggests a general approach for using Rgs genes to identify novel ligands and GPCRs and develop biomarkers and drugs for inflammatory diseases and cancers. Because Rgs regulation of GPCR signaling is ubiquitous during embryonic and childhood development, and adult life, the approach of using Rgs reporter genes to understand PDA is broadly applicable to identifying therapeutic interventions for the treatment of many cancers and disease conditions.

## Material and methods

### Ethical considerations

All experimental procedures were reviewed and approved by the UT Southwestern Medical Center Institutional Animal Care and Use Committee (IACUC protocol: 2016-101480) and conducted in accordance with the UT Southwestern Medical Center IACUC guidelines. All efforts were made to minimize animal suffering.

### Comparative gene expression

Whole exome sequencing genomic data was obtained from the publicly available TCGA (Cancer Genome Atlas) database for all samples of pancreatic ductal adenocarcinoma (PDA). Only samples that had mutational profiling and RNA sequencing data available were included. The mutation annotation format file used was the latest mc3 + caller version created as part of the recent TCGA pan cancer atlas study. RNA sequencing data was batch normalized using RNA seq by Expectation–Maximization (RSEM). Tissue digestion for single cell RNA sequencing, single-cell cDNA library preparation, sequencing, and bioinformatics analyses for single cell RNA sequencing was performed as described previously^37^*.* Seurat package (v3.2.0) was used to generate single cell visualizations. Analysis and figure generation were performed using R statistical software [R Core Team (2018). R: A language and environment for statistical computing. R Foundation for Statistical Computing, Vienna, Austria. URL https://www.R-project.org/].

### Mouse models

KIC;Rgs16::GFP (p48^Cre/+^; Kras^G12D/+^; Cdkn2af/f;Rgs16::GFP) (KIC;Rgs16::GFP ), and Rgs16:GFP (WT) male and female mice were used in this study. Genomic DNA from tail clips were genotyped as described previously^22,52^. In short, PCR primers used were: p48^Cre/+^ (For: 5′-CCTGGAAAATGCTTCTGTCCG-3′; Rev: 5′-CAGGGTGTTATAAGCAATCCC-3′; product: 392 bp), LSL-Kras^G12D^ (For: 5′-CTAGCCACCATGGCTTGAGT-3′; Rev: 5′-TCCGAATTCAGTGACTACAGATG-3′; product: 327 bp) and Cdkn2a^f/f^ (For: 5′-TTGTTGGCCCAGGATGCCGACATC-3′; Rev: 5′-CCAAGTGTGCAAACCCAGGCTCC-3′; product: 145 bp for wild type, 179 bp for *loxP* inserted allele). PCR conditions used were: genomic DNA was denatured at 94 °C for 10 min followed by 33 cycles of 94 °C denaturation (30 s), 60 °C annealing (1 min), and 72 °C elongation (1 min). PCR products were separated via gel electrophoresis in a 1% agarose gel. *Rgs16::GFP expression* was confirmed by blue light excitation of GFP in the brain of newborn pups or in the retina of adult mice. Mice were maintained at a 12-h day, 12-h dark cycle on normal chow ad libitum^53^.

### Caerulein treatment

Pancreatitis was induced in 4 week old adult mice by eight hourly intraperitoneal injections of 0.1 μg/g caerulein (Bachem Americas, Inc., Torrance, CA) for 2 days (i.e. day − 1 and day 0)^54^. Mice were sacrificed at 2, 4, 7, and 14 days post caerulein injections. Bulk RNAseq was prepared from pancreata isolated at day 2, day 4, and day 7 following caerulein treatment. RNAseq analyses were perform as described previously^55^.

### Tissue preparation

Mice were euthanized with CO_2_ asphyxiation; pancreas and intestines were collected, followed by dissection of pancreas with intact HPCD, bile duct, gal bladder, and a segment of the duodenum, washed in PBS (4 °C), and formalin (10%) fixation overnight. Fixed tissue samples were washed in PBS and stored in 70% EtOH (4 °C) until embedded in paraffin, and sectioned (5 μM slices).

### H&E staining and Immunohistochemistry

Pancreatic sections were deparaffinized with xylene and rehydrated to deionized water, stained with hematoxylin and eosin (H&E). Immunohistochemical staining and analysis were performed using anti-GFP (Cell Signaling Technology, Danvers, MA), and anti-Sox9 (Cell Signaling Technology) antibodies. Following deparaffinization with xylene, and antigen retrieval with citrate buffer, endogenous peroxidase activity was inhibited by incubating the tissue sections in 5% H_2_O_2_ for 15 min. The slides were then incubated with 5% normal goat serum (Millipore Sigma, St. Louis, MO) in TBST buffer (tris-buffered saline with 1% tween 20) for 1 h at room temperature. Tissue sections were incubated with primary antibody overnight at 4 °C. Following wash with TBST buffer, tissue sections were incubated for 1 h with horseradish peroxidase conjugated secondary antibody (Vector Laboratories, Beringame, CA) for enzymatic detection or with florescent tagged secondary antibody for immunofluorescence detection. Slides stained with horseradish peroxidase conjugated secondary antibody were development using chromogenic substrate: DAB (Biocare Medical, Pacheco, CA).

### Cell culture

Primary PDA cells were derived from 6 week old KIC;RGS16::GFP mice. Cells were cultured as described previously^22^. In short, cells were grown in Dulbecco’s 72 modified eagle medium (DMEM) (HyClone, Logan, UT), supplemented with 10% Fetal Bovine Serum (FBS) (Sigma-Aldrich, St. Louis, MO) and Penicillin–Streptomycin (P/S) (Millipore Sigma) on rat tail collagen type 1 (BD Biosciences, San Jose, CA) coated plates (0.5 μg/cm^2^) in a humidified incubator at 37 °C and 5% CO_2_. For maintaining, cells were passaged two times a week before reaching complete confluence; washed with calcium and magnesium-free phosphate-buffered saline, and lifted with 0.05% Trypsin–EDTA (HyClone) treatment for up to 10 min in the incubator, and pelleted at 1200 rpm for 3 min. Cells were suspended in fresh medium and plated as required.

### Drug assays

Drugs used for the assays include Trichostatin A (TSA) (APExBIO Technology LLC, Houston, TX) (0, 25, 50, 100, 200, 400, 600, 800 nM), JQ1 (APExBIO Technology LLC) (0, 25, 50,100, 200, 400, 600, 800 nM), Gemcitabine (Gem) (UT Southwestern Pharmacy, Dallas, TX) (0, 6.25, 12.5, 25, 50, 100, and 200 nM), and ISX9 (50 µM). Cells from indicated passage number were seeded into 12 well plates (1 × 10^5^ cells/well) unless otherwise indicated. Optimal cell confluence was considered based on the doubling time. After 24 h, exponentially growing cells were treated with increasing concentrations of TSA and JQ1, alone, in combination, or in combination with Gem, for 72 h.

### Resazurin cell viability assay

Resazurin was used to assess viable cells with active metabolism^56,57^. Resazurin sodium salt (Millipore Sigma) was dissolved at a concentration of 0.4 mg/ml in phosphate buffered saline containing calcium and magnesium, filtered and stored at − 20 °C until use. After cells were treated with the appropriate drug(s), resazurin solution was added to culture medium at a final concentration of 0.02 mg/ml and incubated with cells at 37 °C in CO_2_-incubator for 3 h. Resazurin was reduced by cells with active metabolism to resorufin. Resorufin was detected by *SpectraMax* *Plus* 384 Microplate reader (Molecular Devices, San Jose, CA) using excitation/emission at 560 nm/590 nm.

### Fluorescent microscopy and GFP quantification

Pancreatic expression of Rgs16::GFP in *KIC;Rgs16::GFP* mice was captured and quantified as described^22^. Briefly, a Zeiss Lumar tissue dissection microscope was used to capture the images. Images were captured via a single-channel camera (Hamamatsu Photonics 60-C, 1″, 1×) in 1344 × 1024 resolution with 1 s exposure and 1 × 1 binning, analog gain = 10, and analog offset = 2 settings. Pancreatic fields representing the tumor burden (five or more) of the pancreas were imaged, covering up to 50% of the organ surface area. Images were quantified using NIH ImageJ (https://imagej.nih.gov/ij/; publicly available) software with background subtraction with a radius of 50 pixels. A variable and tight lower threshold was set to eliminate residual background. Intensities of all particles with size ≥ 5 pixels were summed to obtain the total light intensity per image.

GE IN Cell Analyzer 6000 microscope equipped with a Nikon 20 X/0.45 objective was used for GFP expression determination in primary PDA cells. After 72 h of incubation with drug(s), cells were imaged using 405 and 488 lasers lines for Hoechst 33342 and GFP, respectively. Sixteen fields of view per well were captured using a 4-megapixel CMOS camera binned at 2 × 2. Images were analyzed using the GE Analyzer Workstation v3.7.3 software (URL not available for the version used in this paper. URL for currently available version: https://download.cytivalifesciences.com/cellanalysis/download_data/incell/6500/incell_6500_download_page.htm; licensed). Nuclei and cellular compartments were segmented using their respective wavelengths. The mean GFP signal intensity for each cellular object was measured.

### RNA preparation and quantitative RT-PCR

Cells were collected with Trizol (Invitrogen, CA, USA) and total RNA was extracted using the Direct-zol RNA MiniPrep kit (Zymo Research, CA, USA). Following DNase treatment, cDNA was synthesized using SuperScript II Reverse Transcriptase (Invitrogen). Real-time PCR was performed on 10–50 ng of cDNA in triplicate using SsoAdvanced Universal SYBR Green Supermix (Bio-Rad, CA, USA) and Bio-Rad CFX96 Real-Time PCR Detection System. Primers for qPCR were designed using the Primer-BLAST (NCBI Tools) and validated. Data were analyzed using the comparative C_T_ method (2^−ΔΔC^_T_)^58^ using cyclophilin B as a reference gene for normalization. Relative changes in expression of target gene in response to drug treatment were expressed as fold induction compared with the level of expression (given as 1) in non-treated cells.

### Statistical analysis

Data are reported as mean ± SEM. Graphs and their statistical comparisons were performed using GraphPad Prism v7 software [Graphpad Software Inc., CA, USA, https://www.graphpad.com/ (current version); licensed]. Between-subject ANOVAs were used to detect significant changes among multiple groups or conditions followed by tukey’s post-hoc test. CompuSyn synergy software (https://www.combosyn.com/; publicly available) based on the drug combination principles of Chou-Martin^59^ was used for combination index (CI) values quantification. An alpha of *p* < 0.05 was considered statistically significant.

## Supplementary information

Supplementary Figure 1.

Supplementary Figure 2.

Supplementary Figure 3.

Supplementary Legends.

Supplementary Table S1.

## References

[1] Pannala R (2008). Prevalence and clinical profile of pancreatic cancer-associated diabetes mellitus. Gastroenterology.

[2] Rahib L (2014). Projecting cancer incidence and deaths to 2030: The unexpected burden of thyroid, liver, and pancreas cancers in the United States. Cancer Res..

[3] Siegel R, Ma J, Zou Z, Jemal A (2014). Cancer statistics, 2014. CA Cancer J. Clin..

[4] Ben Q (2011). Diabetes mellitus and risk of pancreatic cancer: A meta-analysis of cohort studies. Eur. J. Cancer.

[5] Bosetti C (2012). Cigarette smoking and pancreatic cancer: An analysis from the International Pancreatic Cancer Case-Control Consortium (Panc4). Ann. Oncol..

[6] Siegel RL, Miller KD, Jemal A (2019). Cancer statistics, 2019. CA Cancer J. Clin..

[7] Burris HA (1997). Improvements in survival and clinical benefit with gemcitabine as first-line therapy for patients with advanced pancreas cancer: A randomized trial. J. Clin. Oncol..

[8] Conroy T (2011). FOLFIRINOX versus gemcitabine for metastatic pancreatic cancer. N. Engl. J. Med..

[9] Gourgou-Bourgade S (2013). Impact of FOLFIRINOX compared with gemcitabine on quality of life in patients with metastatic pancreatic cancer: Results from the PRODIGE 4/ACCORD 11 randomized trial. J. Clin. Oncol..

[10] Peddi PF (2012). Multi-institutional experience with FOLFIRINOX in pancreatic adenocarcinoma. JOP.

[11] Collins MA (2012). Oncogenic Kras is required for both the initiation and maintenance of pancreatic cancer in mice. J. Clin. Investig..

[12] Eser S, Schnieke A, Schneider G, Saur D (2014). Oncogenic KRAS signalling in pancreatic cancer. Br. J. Cancer.

[13] Scheffzek K (1997). The Ras-RasGAP complex: Structural basis for GTPase activation and its loss in oncogenic Ras mutants. Science.

[14] Huang H (2014). Oncogenic K-Ras requires activation for enhanced activity. Oncogene.

[15] van Biesen T (1995). Receptor-tyrosine-kinase- and G beta gamma-mediated MAP kinase activation by a common signalling pathway. Nature.

[16] Kahn RA (2014). Is the model of signal amplification by GPCRs/GEFs activating multiple GTPases relevant to a broad spectrum of heterotrimeric and RAS superfamily GTPases?. Cell Logist..

[17] Berman DM, Wilkie TM, Gilman AG (1996). GAIP and RGS4 are GTPase-activating proteins for the Gi subfamily of G protein alpha subunits. Cell.

[18] Hepler JR, Berman DM, Gilman AG, Kozasa T (1997). RGS4 and GAIP are GTPase-activating proteins for Gq alpha and block activation of phospholipase C beta by gamma-thio-GTP-Gq alpha. Proc. Natl. Acad. Sci. U. S. A..

[19] Ross EM, Wilkie TM (2000). GTPase-activating proteins for heterotrimeric G proteins: Regulators of G protein signaling (RGS) and RGS-like proteins. Annu. Rev. Biochem..

[20] Apanovitch DM, Slep KC, Sigler PB, Dohlman HG (1998). Sst2 is a GTPase-activating protein for Gpa1: Purification and characterization of a cognate RGS-Galpha protein pair in yeast. Biochemistry.

[21] Dohlman HG, Song J, Ma D, Courchesne WE, Thorner J (1996). Sst2, a negative regulator of pheromone signaling in the yeast Saccharomyces cerevisiae: Expression, localization, and genetic interaction and physical association with Gpa1 (the G-protein alpha subunit). Mol. Cell Biol..

[22] Ocal O (2015). A rapid in vivo screen for pancreatic ductal adenocarcinoma therapeutics. Dis. Model Mech..

[23] Villasenor A (2010). Rgs16 and Rgs8 in embryonic endocrine pancreas and mouse models of diabetes. Dis. Model Mech..

[24] Ducasse M, Brown MA (2006). Epigenetic aberrations and cancer. Mol. Cancer.

[25] Marks PA, Dokmanovic M (2005). Histone deacetylase inhibitors: Discovery and development as anticancer agents. Expert Opin. Investig. Drugs.

[26] Fournel M (2002). Sulfonamide anilides, a novel class of histone deacetylase inhibitors, are antiproliferative against human tumors. Cancer Res..

[27] Batova A (2002). The histone deacetylase inhibitor AN-9 has selective toxicity to acute leukemia and drug-resistant primary leukemia and cancer cell lines. Blood.

[28] Nervi C (2001). Inhibition of histone deacetylase activity by trichostatin A modulates gene expression during mouse embryogenesis without apparent toxicity. Cancer Res..

[29] Vigushin DM (2001). Trichostatin A is a histone deacetylase inhibitor with potent antitumor activity against breast cancer in vivo. Clin. Cancer Res..

[30] Sahai V (2014). BET bromodomain inhibitors block growth of pancreatic cancer cells in three-dimensional collagen. Mol. Cancer Ther..

[31] Fritsche P (2009). HDAC2 mediates therapeutic resistance of pancreatic cancer cells via the BH3-only protein NOXA. Gut.

[32] Cai MH (2018). Depletion of HDAC1, 7 and 8 by histone deacetylase inhibition confers elimination of pancreatic cancer stem cells in combination with gemcitabine. Sci. Rep..

[33] Klieser E (2015). Role of histone deacetylases in pancreas: Implications for pathogenesis and therapy. World J. Gastrointest. Oncol..

[34] Manzotti G, Ciarrocchi A, Sancisi V (2019). Inhibition of BET proteins and histone deacetylase (HDACs): Crossing roads in cancer therapy. Cancers (Basel)..

[35] Xie F (2018). The BET inhibitor I-BET762 inhibits pancreatic ductal adenocarcinoma cell proliferation and enhances the therapeutic effect of gemcitabine. Sci. Rep..

[36] von Figura G, Morris JPT, Wright CV, Hebrok M (2014). Nr5a2 maintains acinar cell differentiation and constrains oncogenic Kras-mediated pancreatic neoplastic initiation. Gut.

[37] Hosein AN (2019). Cellular heterogeneity during mouse pancreatic ductal adenocarcinoma progression at single-cell resolution. JCI Insight.

[38] Aguirre AJ (2003). Activated Kras and Ink4a/Arf deficiency cooperate to produce metastatic pancreatic ductal adenocarcinoma. Genes Dev..

[39] Dioum EM (2011). A small molecule differentiation inducer increases insulin production by pancreatic beta cells. Proc. Natl. Acad. Sci. U. S. A..

[40] Finnin MS (1999). Structures of a histone deacetylase homologue bound to the TSA and SAHA inhibitors. Nature.

[41] Li GC, Zhang X, Pan TJ, Chen Z, Ye ZQ (2006). Histone deacetylase inhibitor trichostatin A inhibits the growth of bladder cancer cells through induction of p21WAF1 and G1 cell cycle arrest. Int. J. Urol..

[42] Horing E (2013). The histone deacetylase inhibitor trichostatin a promotes apoptosis and antitumor immunity in glioblastoma cells. Anticancer Res..

[43] Donadelli M (2007). Synergistic inhibition of pancreatic adenocarcinoma cell growth by trichostatin A and gemcitabine. Biochim. Biophys. Acta.

[44] Wang B (2018). FBP1 loss contributes to BET inhibitors resistance by undermining c-Myc expression in pancreatic ductal adenocarcinoma. J. Exp. Clin. Cancer Res..

[45] Wieczorek M (2016). The synthetic diazonamide DZ-2384 has distinct effects on microtubule curvature and dynamics without neurotoxicity. Sci. Transl. Med..

[46] Hezel AF, Kimmelman AC, Stanger BZ, Bardeesy N, Depinho RA (2006). Genetics and biology of pancreatic ductal adenocarcinoma. Genes Dev..

[47] Vigil D, Cherfils J, Rossman KL, Der CJ (2010). Ras superfamily GEFs and GAPs: Validated and tractable targets for cancer therapy?. Nat. Rev. Cancer.

[48] Krah NM (2015). The acinar differentiation determinant PTF1A inhibits initiation of pancreatic ductal adenocarcinoma. Elife.

[49] Cobo I (2018). Transcriptional regulation by NR5A2 links differentiation and inflammation in the pancreas. Nature.

[50] Langdon CG (2017). Combinatorial screening of pancreatic adenocarcinoma reveals sensitivity to drug combinations including bromodomain inhibitor plus neddylation inhibitor. Mol. Cancer Ther..

[51] Mazur PK (2015). Combined inhibition of BET family proteins and histone deacetylases as a potential epigenetics-based therapy for pancreatic ductal adenocarcinoma. Nat. Med..

[52] Zolghadri Y (2018). Malnutrition in pancreatic ductal adenocarcinoma (PDA): Dietary pancreatic enzymes improve short-term health but stimulate tumor growth. Am. J. Pathol..

[53] Huang J (2006). Feeding and fasting controls liver expression of a regulator of G protein signaling (Rgs16) in periportal hepatocytes. Comp. Hepatol..

[54] Flandez M (2014). Nr5a2 heterozygosity sensitises to, and cooperates with, inflammation in KRas(G12V)-driven pancreatic tumourigenesis. Gut.

[55] Hoang CQ (2016). Transcriptional maintenance of pancreatic acinar identity, differentiation, and homeostasis by PTF1A. Mol. Cell Biol..

[56] Nociari MM, Shalev A, Benias P, Russo C (1998). A novel one-step, highly sensitive fluorometric assay to evaluate cell-mediated cytotoxicity. J. Immunol. Methods.

[57] Rampersad SN (2012). Multiple applications of Alamar Blue as an indicator of metabolic function and cellular health in cell viability bioassays. Sensors (Basel).

[58] Kurrasch DM, Huang J, Wilkie TM, Repa JJ (2004). Quantitative real-time polymerase chain reaction measurement of regulators of G-protein signaling mRNA levels in mouse tissues. Methods Enzymol..

[59] Chou TC, Martin N (2007). CompuSyn Software for Drug Combinations and for General Dose-Effect Analysis, and User's Guide.

[60] Zhang W (2018). Small cell lung cancer tumors and preclinical models display heterogeneity of neuroendocrine phenotypes. Transl. Lung Cancer Res..

[61] Cerami E (2012). The cBio cancer genomics portal: An open platform for exploring multidimensional cancer genomics data. Cancer Discov..

[62] Gao J (2013). Integrative analysis of complex cancer genomics and clinical profiles using the cBioPortal. Sci Signal.

