# Supplementary figures and images for: Concerted cell and in vivo screen for pancreatic ductal adenocarcinoma (PDA) chemotherapeutics
Source: Sci Rep. 2020 Nov 26;10:20662. doi: 10.1038/s41598-020-77373-8 (PMC7693321; doi:10.1038/s41598-020-77373-8)

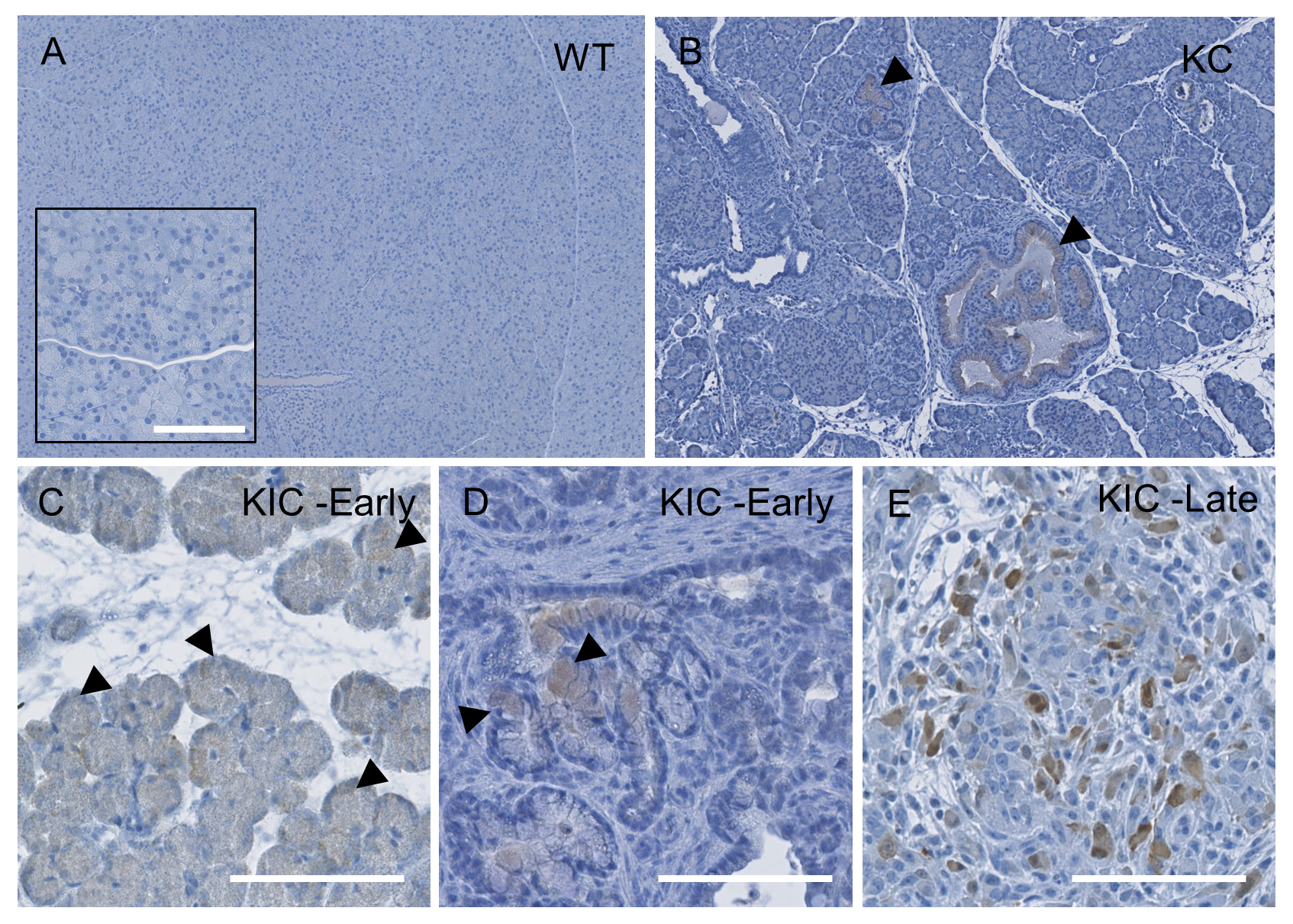

Supplement: Supplementary file 1 — Supplementary Figure 1. [file 41598_2020_77373_MOESM1_ESM.tif]

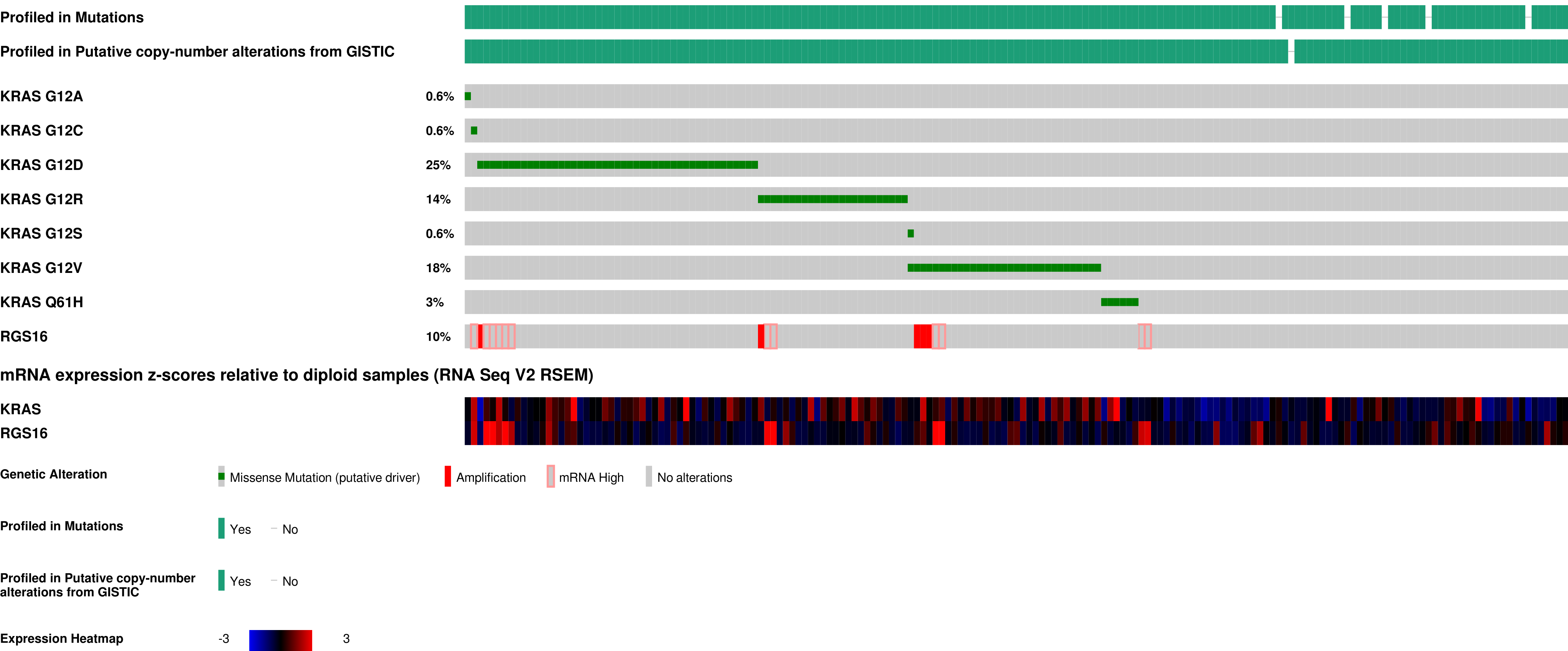

Supplement: Supplementary file 2 — Supplementary Figure 2. [file 41598_2020_77373_MOESM2_ESM.tiff]

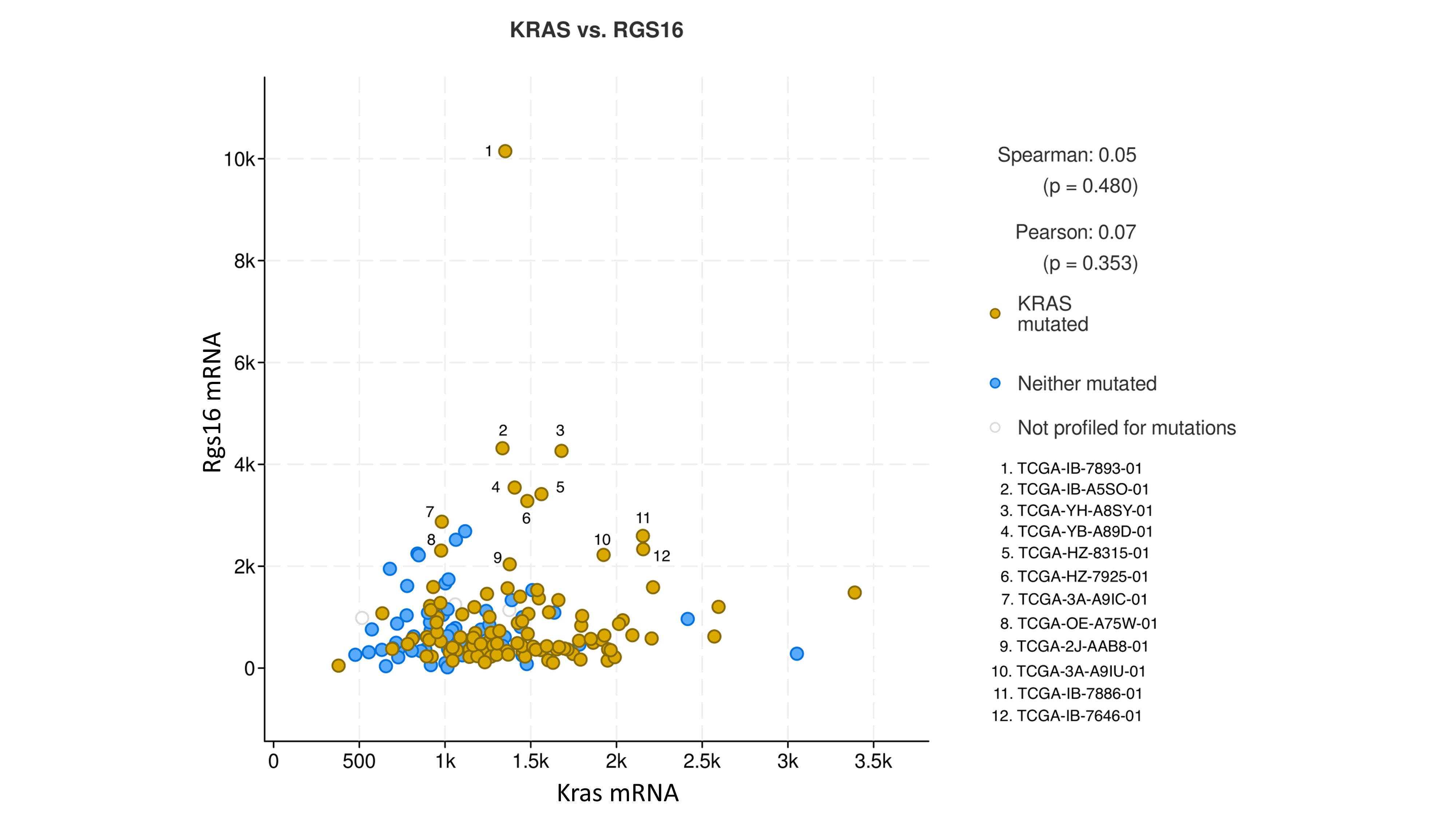

Supplement: Supplementary file 3 — Supplementary Figure 3. [file 41598_2020_77373_MOESM3_ESM.tif]
