# Supplementary material for: Concerted cell and in vivo screen for pancreatic ductal adenocarcinoma (PDA) chemotherapeutics
Source: Sci Rep. 2020 Nov 26;10:20662. doi: 10.1038/s41598-020-77373-8 (PMC7693321; doi:10.1038/s41598-020-77373-8)
Supplement: Supplementary file 4 — Supplementary Legends. [file 41598_2020_77373_MOESM4_ESM.docx]

**Title: Concerted cell and *in vivo* screen for pancreatic ductal adenocarcinoma (PDA) chemotherapeutics**

Somayeh Layeghi-Ghalehsoukhteh^1,2^**^#^** , Shreoshi Pal Choudhuri^1^**^#^** , [Ozhan Ocal](http://dmm.biologists.org/search?author1=Ozhan+Ocal&sortspec=date&submit=Submit)^1,3^**^#^**[, Yalda Zolghadri](http://dmm.biologists.org/search?author1=Yalda+Zolghadri&sortspec=date&submit=Submit)^1,2^, Victor Pashkov^1^, Hanspeter Niederstrasser^4^, Bruce A. Posner^4^, Havish S. Kantheti^1,5^, Ana C. Azevedo-Pouly^6^, Huocong Huang^7^, Luc Girard ^1,7,8^, Raymond J. MacDonald^9^, Rolf A. Brekken ^7,10^ and [Thomas M. Wilkie](http://dmm.biologists.org/search?author1=Thomas+M.+Wilkie&sortspec=date&submit=Submit)^1*^

**#** These authors contributed equally

Laboratory of Origin: T.M.W*, Corresponding Author

1. Department of Pharmacology, UT Southwestern Medical Center, 6001 Forest Park Drive, Dallas, TX 75390, USA

2. Department of Basic Science, School of Veterinary Medicine, Shiraz University, Shiraz, Iran

3. Department of Molecular Biology and Genetics, Bilkent University, Ankara, 06800, Turkey

4. Department of Biochemistry, UT Southwestern Medical Center, 5323 Harry Hines Blvd, Dallas, TX 75390, USA

5. Cancer Discovery (CanDisc) Group, UT Southwestern Medical Center, 6001 Forest Park Drive, Dallas, TX 75390, USA

6. Department of Surgery, University of Arkansas for Medical Sciences, Little Rock, Arkansas, United States of America

7. Hamon Center for Therapeutic Oncology Research, University of Texas Southwestern Medical Center, Dallas, TX, USA

8. Simmons Comprehensive Cancer Center, University of Texas Southwestern Medical Center, Dallas, TX, 75390, USA

9. Department of Molecular Biology, UT Southwestern Medical Center, 5323 Harry Hines Blvd, Dallas, TX 75390, USA

10. Department of Surgery, University of Texas Southwestern Medical Center, Dallas, TX, USA

***Corresponding Author:** Thomas M. Wilkie, Department of Pharmacology, UT Southwestern Medical Center, 6001 Forest Park Drive, Dallas, TX 75390, United States. Office: (214) 645-6175; Cell (214) 394-8275

email: [Thomas.Wilkie@UTSouthwestern.edu](mailto:Thomas.Wilkie@UTSouthwestern.edu)

**Figure S1: Rgs16::GFP is expressed in PanIN lesions and PDA in KC and KIC mice**. Immunohistochemical staining showing GPF expression in the pancreas sections of KC; Rgs16::GFP and KIC;RGS16::GFP mice. No GFP staining was detected in (A) normal, healthy Rgs16::GFP mice. GFP staining was detected in (B) PanIN lesions in KC (arrowhead), (C) dedifferentiated pancreas in early KIC (arrowhead), (D) PanIN lesions in early KIC (arrowhead), and in (E) late KIC tumor (cells stained brown). Scale bars are 100 µM.

**Figure S2: Relative Rgs16 mRNA expression in human PDA tumor samples with WT or oncogenic Kras.** Oncoprint from cBioPortal^61, 62^ shows a custom correlation analysis for KRAS mutant vs. RGS16 and KRAS Not Altered vs. RGS16. KRAS mutant vs. RGS16 (Spearman: 0.08607623, p-value = 0.3707, Pearson: 0.07069626 , p-value = 0.463). KRAS Not Altered vs. RGS16 (Spearman: 0.07382872, p-value = 0.5518 Pearson: -0.03564976 , p-value = 0.7746). Relative expression heat map: red is high, blue is low expression. R (v4.0.2) software (<https://www.r-project.org/>; publicly available) was used for data analysis and visualization.

**Figure S3. Rgs16 and Kras mRNA expression comparison in human PDA tumor samples with WT or oncogenic Kras.** Tissue samples from the cBioPortal database^61, 62^. mRNA expression, RSEM (batch normalized from Illumina HiSeq_RNASeqV2). Gold circles, oncogenic Kras mutations; blue circles, Kras not altered; open circles, not determined. TCGA sample number indicated for all 12 samples with oncogenic Kras mutations and Rgs16 mRNA expression above 2k RSEM. R (v4.0.2) software (<https://www.r-project.org/>; publicly available) was used for data analysis and visualization.

**Table S1: Rgs16 mRNA expression in human cancer cell lines with WT or oncogenic Kras.** All RNASeq data were available for human cancer cell lines from the TCGA database, except lung samples from 81 resected SCLC tumors and for 70 SCLC cell lines that we established and characterized in the Minna laboratory^60^. Cell lines from 9 cancer types were analyzed; 157 oncogenic Kras cell lines (G12X, G13X, Q61X), 459 WT Kras cell lines (# oncogenic, # WT Kras) are from pancreas (45 onco, 4 WT Kras), lung (NSCLC; 44, 98), biliary track (7, 14), breast (2, 52), endometrium (4, 20), lymphoid (11, 169), colon (30, 25), ovary (6, 47), and stomach (8, 30). Rgs16 mRNA expression is indicated relative to Kras expression as a log ratio (dark blue is high, white is low; Rgs16/Kras), with the associated T-test value. Kras allele genotypes are indicated. Cell lines are identified by CCLE or Minna lab name.
